# A study of acoustic characteristics of voluntary expiratory sounds produced before and immediately after swallowing

**DOI:** 10.1038/s41598-021-04624-7

**Published:** 2022-01-14

**Authors:** Shoma Hattori, Shinji Nozue, Yoshiaki Ihara, Koji Takahashi

**Affiliations:** Showa University, Tokyo, Japan

**Keywords:** Medical research, Risk factors

## Abstract

To evaluate the expiratory sounds produced during swallowing recorded simultaneously with videofluorographic examination of swallowing (VF) using fast Fourier transform (FFT), and to examine the relationship between dysphagia and its acoustic characteristics. A total of 348 samples of expiratory sounds were collected from 61 patients with dysphagia whose expiratory sounds were recorded during VF. The VF results were evaluated by one dentist and categorized into three groups: safe group (SG), penetration group (PG), and aspiration group (AG). The duration and maximum amplitude of expiratory sounds produced were measured as the domain characteristics on the time waveform of these sounds and compared among the groups. Time window-length appropriate for FFT and acoustic discriminate values (AD values) of SG, PG, and AG were also investigated. The groups were analyzed using analysis of variance and Scheffé's multiple comparison method. The maximum amplitude of SG was significantly smaller than those of PG and AG. The mean duration in SG (2.05 s) was significantly longer than those in PG (0.84 s) and AG (0.96 s). The AD value in SG was significantly lower than those in PG and AG. AD value detects penetration or aspiration, and can be useful in screening for dysphagia.

## Introduction

Japan is a super-aging society and has one of the longest life expectancies in the world. Dysphagia is a common pathologic condition in elderly people with or without geriatric diseases, such as stroke, Parkinson’s disease, and neurological disorders. Therefore, dysphagia assessments are widely performed to evaluate ingestion in several settings, such as medical fields and nursing facilities in Japan. The gold standards for the diagnosis of dysphagia are videoendoscopic examination of swallowing (VE) and videofluorographic examination of swallowing (VF). VE is probably the most frequently used tool for objective dysphagia assessment. It allows for the evaluation of the efficacy and safety of swallowing, determination of appropriate feeding strategies, and assessment of the efficacy of different swallowing maneuvers ^1,2^. However, the demerit of VE is that patients feel uncomfortable with a fiber passing through the nasal cavity during the examination. On the other hand, VF can be used to investigate the entire swallowing activity, from the oral stage to the esophageal stage^2,3^. The pathological, morphological, and functional aspects of swallowing are evaluated using VF findings. However, the inspection sites and study designs using VF have been limited because of radiation exposure. There are many alternative assessment methods for detecting dysphagia, such as cervical auscultation (CA)^4–6^, repetitive saliva swallowing test^7^, and water swallowing test^7,8^. CA has been used for detecting dysphagia and is a noninvasive screening tool for assessing aspiration, penetration, and pharyngeal retention^9–11^. It has been used widely in the clinical setting, including medical fields and nursing facilities^12^. Moreover, CA is used at meal rounds in nursing facilities and in home-visit medical care.

Several studies have evaluated the diagnostic accuracy of CA using subjective evaluation^4,6,13,14^. Studies focusing on acoustic data detected from the neck during swallowing attempts using VF or VE, reported that the diagnostic accuracy of CA for penetration or aspiration had a sensitivity of 62–94% and a specificity of 50–100%^13–15^. However, some studies have focused on the diagnostic accuracy of CA through objective evaluation. Hirano et al. analyzed the acoustic signals of expiratory sounds recorded by an accelerometer by octave band analysis, and reported that the 125.0-Hz band was the critical band for detecting dysphagia^16^. Yamashita et al. analyzed the acoustic signals of expiratory sounds recorded using a microphone by one-third octave band analysis, compared them with VF images, and calculated the value obtained by subtracting the averaged level of the 1000.0-Hz reference band of the expiratory sounds’ central frequency from the sound level of 125.0-Hz central frequency. They reported that the consistency rate of diagnosis for penetration/aspiration using expiratory sounds of 85.4% based on VF findings^17^. These studies investigated the relevance of the frequency characteristics for expiratory sounds and VF images using one-third octave band analysis or one octave band analysis to approach the auditory perception of humans. On the other hand, narrow-band analysis using the fast Fourier transform (FFT) can be used to analyze the characteristics of expiratory sounds in more detail than one-third octave band analysis or one octave band analysis. The aim of this study was to analyze expiratory sounds produced during swallowing recorded simultaneously with VF using the FFT focusing on the low frequency band, and to examine the relationship between the dysphagia and the acoustic characteristics of expiratory sounds.

## Methods

### Subjects

Patients with dysphagia and those who complained of dysphagic symptoms and underwent VF in the Department of Oral Rehabilitation, Showa University Dental Hospital were included in the study. The exclusion criteria were as follows: 1) patients wearing tracheal cannula; 2) patients who could not follow the instructions; 3) patients with fatigue, fever, and/or any other poor physical conditions that might influence swallowing function; and 4) patients who could not exhale constantly.

### Compliance with ethical standards

All study protocols and procedures were conducted in accordance with the ethical guidelines and in compliance with the Declaration of Helsinki. Each subject gave written informed consent and the ethics committee of Showa University School of Dentistry granted approval for this study (no. SUDH0063).

### Acquisition of acoustic data

**Figure 1:**
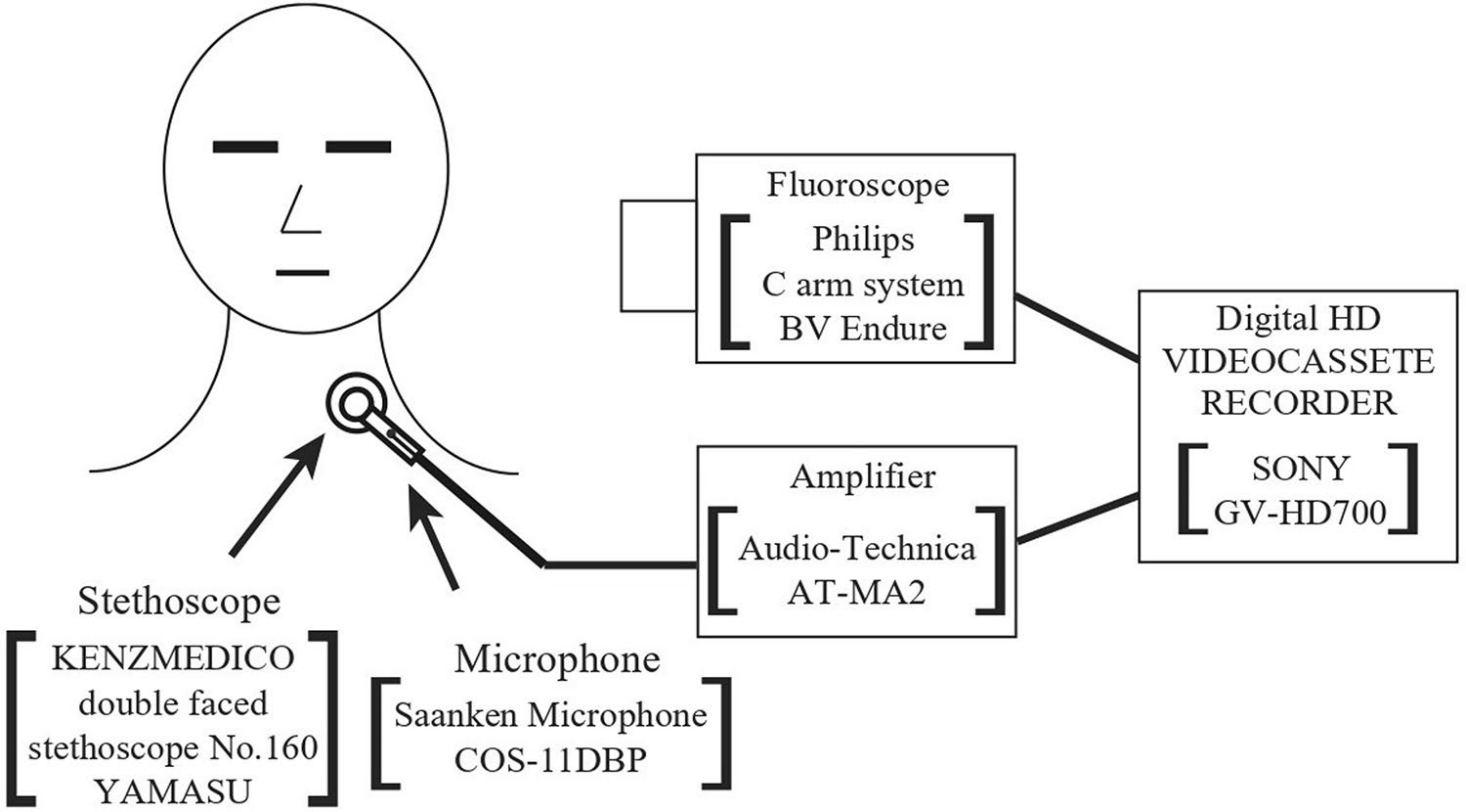
Detecting and recording system of swallow-related sounds. The diaphragm chest piece of a double-faced stethoscope connected to a short tube with an inserted microphone was attached to the site over the lateral border of the trachea immediately inferior to the cricoid cartilage. The detected acoustic signals were amplified, digitally converted with a 48-kHz sampling rate, and recorded with videofluorographic (VF) images into DVCAM tape through digital HD videotape recorder.

At the same time as VF imaging, exhalation sounds were recorded. Before VF, the patient’s neck was cleaned with an alcohol pad, and the diaphragm chest piece of a double-faced stethoscope connected to a short tube with an inserted electret condenser microphone (Sanken Co., COS-11D-BP, Japan) was attached to a site over the lateral border of the trachea immediately inferior to the cricoid cartilage using a 1 cm^2^ piece of double-sided adhesive paper tape^17^ (Fig. 1). Moreover, residual secretions in the oral cavity, pharynx, and larynx of the patients were removed either by strong voluntary cough or by forced expiration. After an auditory impression by one dentist determined that the oral, pharyngeal, and laryngeal residues had been removed, the patients were instructed to practice exhaling three times with constant force. During VF, the patients swallowed test foods containing barium sulfate according to their swallowing ability. The patients were asked to exhale three times with constant force before the sample was inserted into the mouth and again three times immediately after swallowing the whole test sample. The detected acoustic signals, including expiratory sounds were amplified, digitally converted with 48 kHz sampling rate, and the number of quantization bits was 16 bits in wave file format. The characteristic of the electret condenser microphone (Sanken Co., COS-11D-BP, Japan) was flat from over 200 Hz of the whole.

### Classification of subjects based on VF findings

The VF findings were analyzed by one dentist, who has been working in dysphagia rehabilitation for more than 10 years and was certified by the Japanese Society of Dysphagia Rehabilitation, and categorized into three groups: without penetration or aspiration (safe group: SG), with penetration (penetration group: PG), and with aspiration (aspiration group: AG).

### Sound analysis

The maximum amplitude value of the expiratory sounds was measured by Multi speech 3000® (Pentax, Tokyo, Japan). The duration of expiratory sounds obtained from acoustic data were measured by auditory perception by one dentist. The expiratory sounds and noise were excluded.

**Figure 2:**
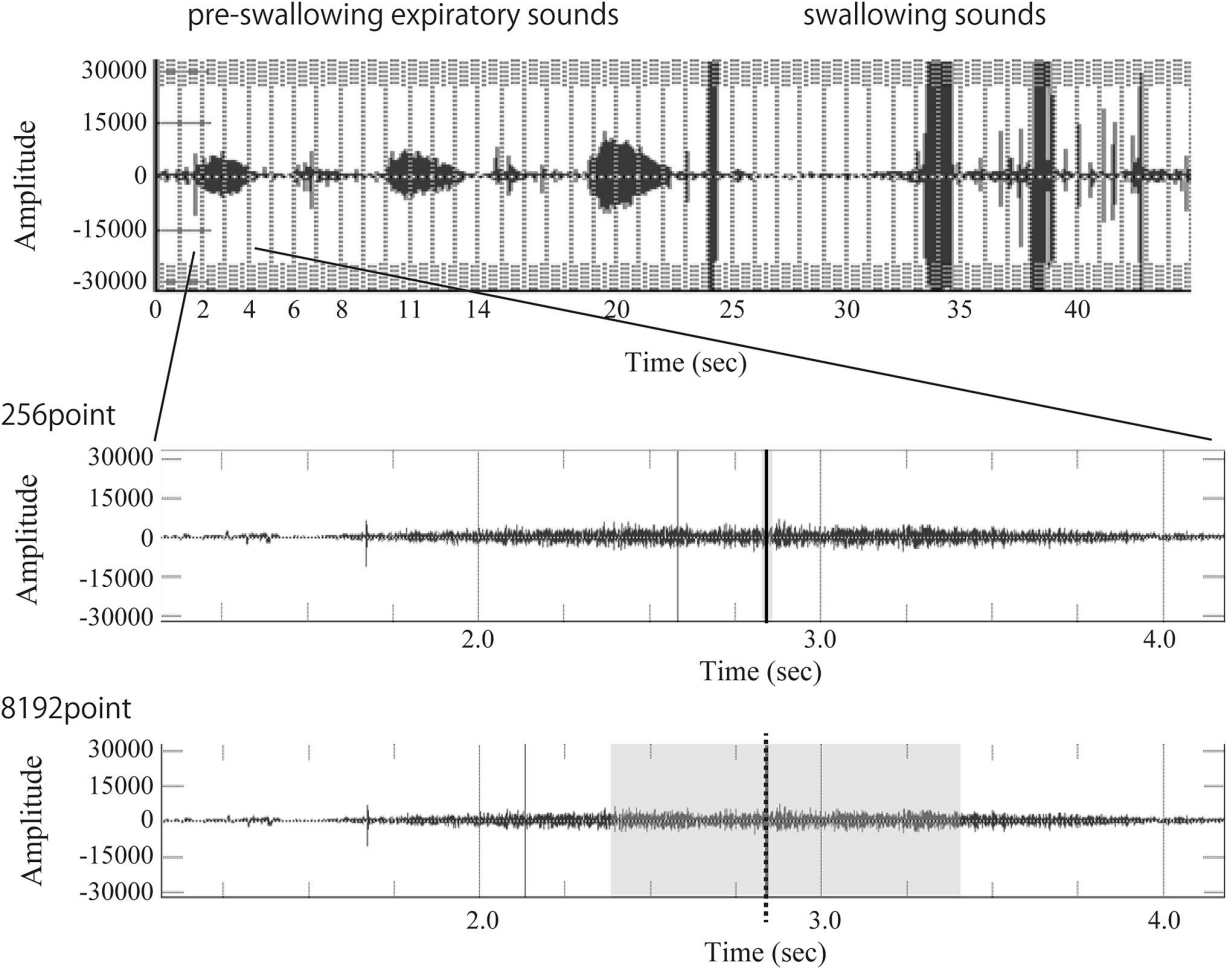
Expiratory sounds and the Hamming window. An example of windowing of 8192 points using the Hamming window of the expiratory sound before swallowing. The three peaks on the left side of the upper figure are the expiratory sounds, and in the lower figure, the leftmost peak is the center of the window.

**Figure 3:**
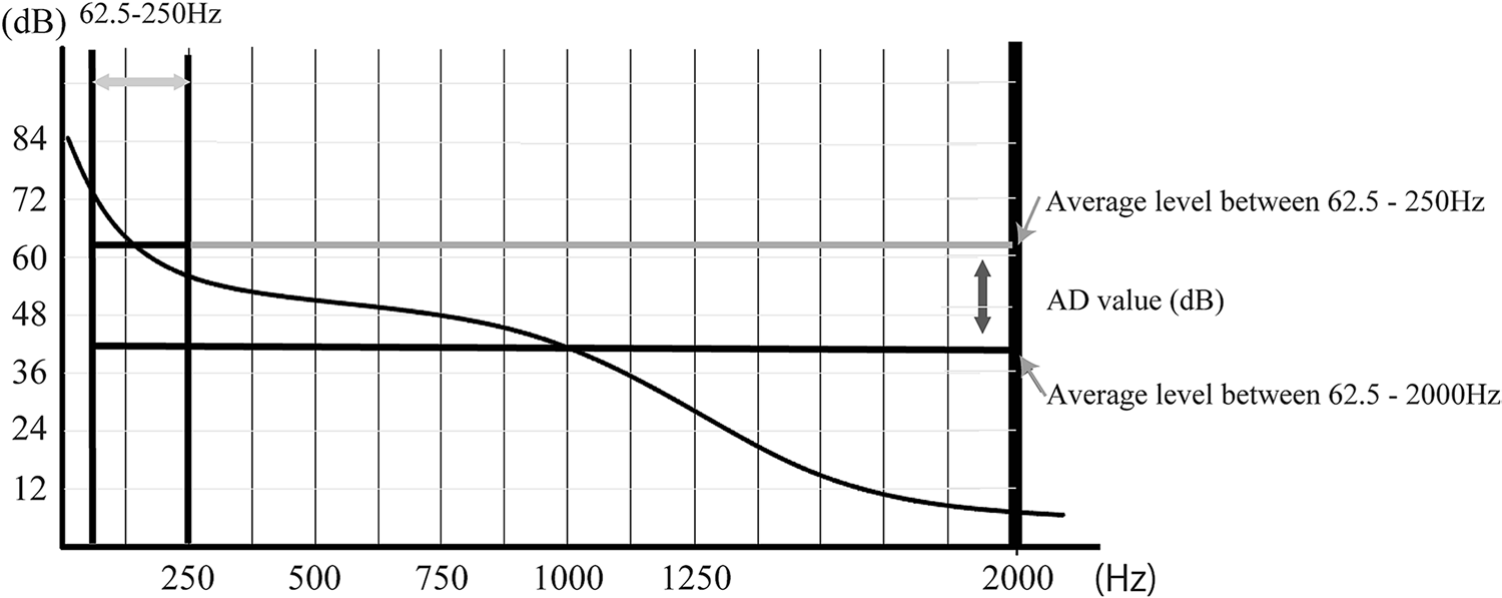
FFT analysis and calculation of AD values. The method to calculate AD values. Calculate the average value of 62.5–250 Hz and 62.5–2000 Hz bands. Then, subtract the average of 62.5–2000 Hz from the average of 62.5–250 Hz band. This value is defined as the AD value.

FFT was used for sound analysis. In the analysis using FFT, it is necessary to use an appropriate sampling rate, because the use of an excessively high sampling rate will affect the frequency band and analysis time. For evaluation of CA, it was considered appropriate to use a frequency range from 62.5 Hz (slightly lower than the human audible range) to 4 kHz (the middle range). The wave files simultaneously recorded with VF images were analyzed at a sampling rate of 48 kHz; the number of quantization bits was 16 bits. After downsampling the wave files to a sampling rate of 8 kHz, we analyzed them using Multi speech 3000®. Expiratory sound before swallowing (ESBS) and expiratory sound after swallowing (ESAS) obtained from the wave files recorded simultaneously with the VF images were Hamming-windowed using four length-of-time windows, including acoustic signals with the maximum amplitude and transformed into frequency waveforms using FFT. The lengths of the time windows were 256 (0.032 s), 1024 (0.128 s), 2048 (0.256 s), and 8192 (1.024 s) (Fig. 2). There were differences in the length of the recording environment and the volume of the voluntary expiratory sounds for each subject. Therefore, normalization was performed by subtracting the average amplitude of the overall frequency band (62.5–2000 Hz) from the average amplitude of the lower frequency band (62.5–250 Hz). The following equation was used to find the average amplitude values for the low and total frequency bands (Fig. 3). The mean dB of each frequency band was obtained by

$$L = 10\log 1/n(10^{{(0.1L_{1} )}} + 10^{{(0.1L_{2} )}} + 10^{{(0.1L_{3} )}} + 10^{{(0.1L_{n} )}} .$$

The acoustic discriminant vale (AD value) was calculated by subtracting the average volume of the total frequency band from the average volume of the low frequency band.

### Statistical analyses

The maximum amplitude value, duration of expiratory sounds, and AD values for ESBS and ESAS in SG, PG, and AG were analyzed using analysis of variance (ANOVA) and Scheffe’s multiple comparison method. Results were accepted as statistically significant at the probability level of 5%. Data were analyzed using SPSS (ver.26.0 SPSS, Chicago, IL, USA).

## Results

### Details of subjects

Sixty-one patients (44 men) with dysphagia were included in this study. The details of the subjects (sex, age, and primary cause of dysphagia) are shown in Table 1. A total of 348 expiratory sound samples were collected in this study. Thirty samples were excluded from the analysis due to reasons, such as clothing abrasion and digital noise.

**Table 1:** AD values of PG and AG were significantly.

		SG	PG	AG	Total
Subjects	N (male)	18(10)	23(17)	20(17)	61(44)
Age	Mean	66.8 $\pm$ 24.4	75.6 $\pm$ 8.1	72.2 $\pm$ 10.3	71.9 $\pm$ 15.8
Underlying	Stroke	1	5	7	13
	Head and neck		15	11	26
	Psychological	4			4
	Dementia	1		1	2
	Disuse syndrome	3			3
	Others	9	3	1	13

### Classification of subjects

Subjects were divided into three groups based on VF findings. SG included 18, PG included 23, and AG included 20 subjects; SG included 91, PG included 148, and AG included 109 expiratory sounds samples.

### Results of the maximum amplitude values for ESBS

**Figure 4:**
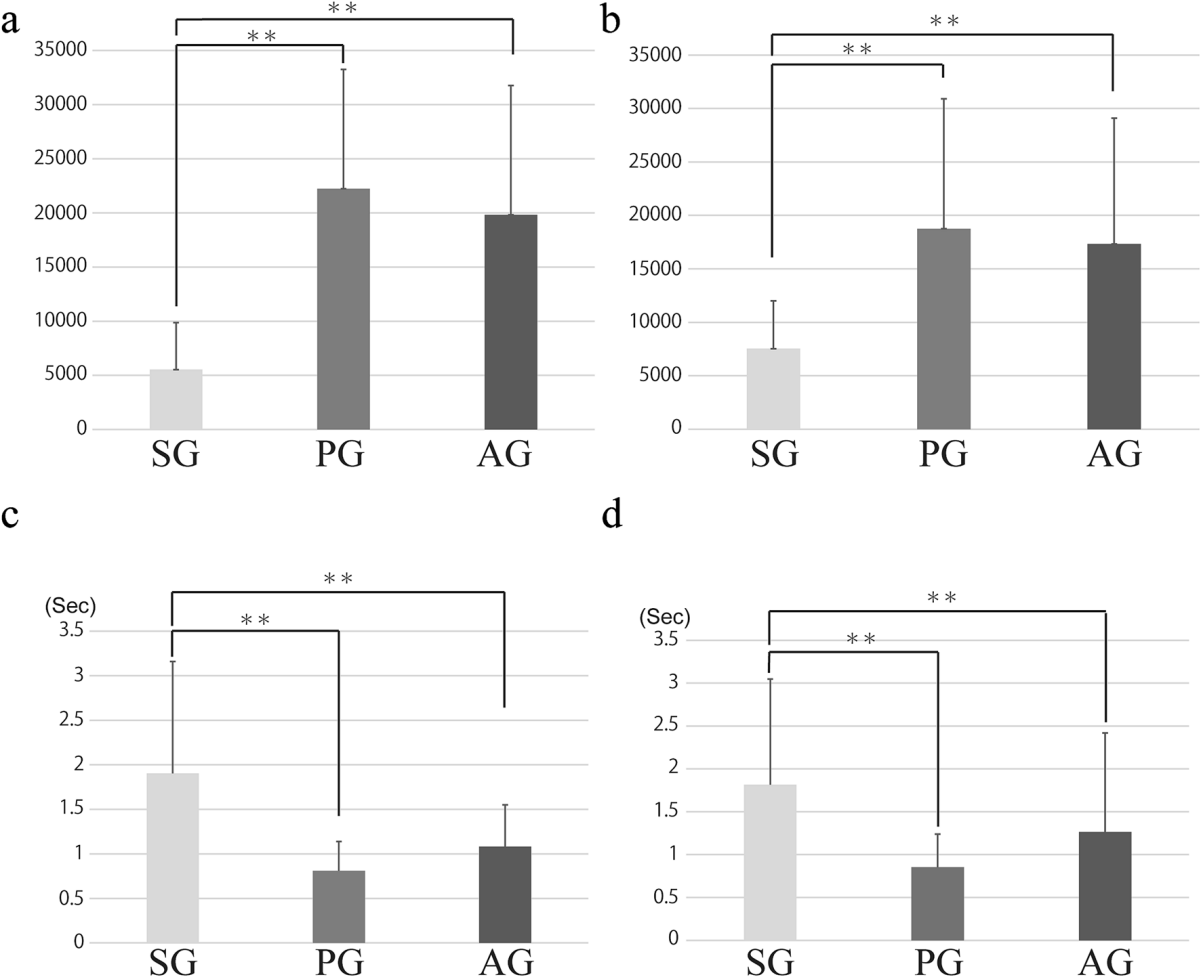
(**a**): The length of SG for ESBS was significantly longer than those of PG and SG. (**b**): The length of SG for ESAS was significantly longer than those of PG and SG. (**c**): The amplitude value of SG for ESBS was significantly lower than those of PG and SG. (**d**): The amplitude value of SG for ESAS was significantly lower than those of PG and SG.

The maximum amplitude values in SG, AG, and PG were 5521.28 (SD = 4335.64), 22231.12 (SD = 11019.50), and 19815.61 (SD = 11953.14), respectively (Table 2). The maximum amplitude in SG was significantly lower than those in PG and AG (Fig. 4).

**Table 2:** Amplitude of expiratory sounds.

	Mean ± SD
**Amplitude of expiratory sounds**	
ESBS SG	5521.28 $\pm$ 4435.64
PG	22231.12 $\pm$ 11019.50
AG	19815.61 $\pm$ 11953.14
ESAS SG	7538.24 $\pm$ 4460.23
PG	18751.82 $\pm$ 12159.01
AG	17336.75 $\pm$ 11765.83
**Duration of expiratory sound (s)**	
ESBS SG	1.90 $\pm$ 1.26
PG	0.81 $\pm$ 0.33
AG	1.08 $\pm$ 0.47
ESAS SG	1.82 $\pm$ 1.23
PG	0.85 $\pm$ 0.39
AG	1.26 $\pm$ 1.16

### Results of the maximum amplitude values for ESAS

The maximum amplitude value in SG, PG, and AG was 7538.24 (SD = 4460.23), 18751.82 (SD = 12159.01), and 17336.75 (SD = 11765.83), respectively (Table 2). The maximum amplitude in SG was significantly lower than those in PG and AG. (Fig. 4).

### Results of the duration about expiratory sounds for ESBS

The mean of the duration of all expiratory sounds was 1.20 s (SD = 0.87). The mean of the duration of all expiratory sounds in SG, PG, and AG was 1.90 s (SD = 1.26), 0.81 s (SD = 0.33), and 1.08 s (SD = 0.47), respectively (Table 2). The mean of the duration of all expiratory sounds in SG was significantly longer than those in PG and AG (*p* = 0.000) (Fig. 4).

### Results of the duration of expiratory sounds for ESAS

The mean of the length of all expiratory sounds was 1.20 s (SD = 0.96). The mean of the length of all expiratory sounds in SG, PG, and AG was 1.82 s (SD = 1.23), 0.85 s (SD = 0.39), and 1.26 s (SD = 1.16), respectively (Table 2). The mean of the length of all expiratory sounds in SG was significantly longer than those in PG and AG (*p* < 0.0001) (Fig. 4).

### Results of the AD values for ESBS

In SG, the AD values for ESBS acquired using the 256, 1024, 2048, and 8192-points time windows were − 3.71 (SD = 5.00), − 2.84 (SD = 5.34), − 2.16 (SD = 5.67), and − 1.60 (SD = 4.96), respectively. In PG, the AD values for ESBS acquired using the 256, 1024, 2048, and 8192-points time windows were 2.38 (SD = 4.88), 1.42 (SD = 5.24), 2.18 (SD = 4.95), and 2.96 (SD = 4.68), respectively. In AG, the AD values for ESBS acquired using the 256, 1024, 2048, and 8192-points time windows were 2.78 (SD = 6.68), 4.00 (SD = 4.93), 4.82 (SD = 4.72), and 5.07 (SD = 4.72), respectively.

**Figure 5:**
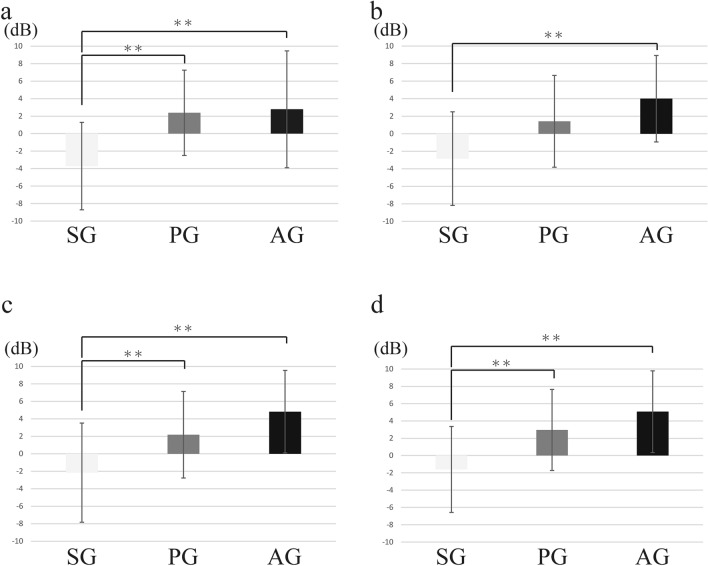
Result of ESBS. (**a**): At 256 points, the AD values of PG and AG were significantly higher than those of SG. (**b**): At 1024 points, AD values of AG were significantly higher than those of SG; however, there was no significant difference between SG and PG. (**c**): At 2048 points, the AD values of PG and AG were significantly higher than those of SG. (**d**): At 8192 points, the AD values of PG and AG were significantly higher than those of SG. (* = p < 0.05, ** = p < 0.01).

Regarding the results from the 256-, 2048-, and 8192-point time windows, the AD values of SG were significantly lower than those of PG (*p* = 0.001, *p* < 0.0001, and *p* = 0.003) and AG (*p* < 0.0001, *p* < 0.0001, and *p* < 0.0001). Regarding the results from the 1024-point time window, the AD value in SG was noted to be significantly lower than that in AG (*p* < 0.001); however, there was no significant difference compared with that in PG (*p* = 0.116) (Fig. 5).

### Results of the AD values for ESAS

**Figure 6:**
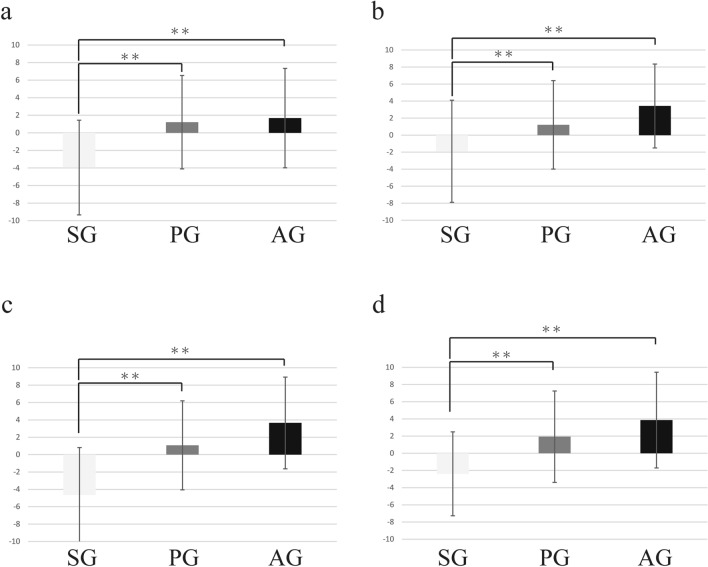
Result of ESAS. (**a**): At 256 points, the AD values of PG and AG were significantly higher than those of SG. (**b**): At 1024 points, the AD values of PG and AG were significantly higher than those of SG. (**c**): At 2048 points, the AD values of PG and AG were significantly higher than those of SG. d: At 8192 points, the AD values of PG and AG were significantly higher than those of SG. (* = *p* < 0.05, ** = *p* < 0.01).

In SG, the AD values for ESAS acquired in the 256, 1024, 2048, and 8192-points time windows were 3.95 (SD = 5.39), -1.89 (SD = 6.00), -4.64 (SD = 5.46), and -2.39 (SD = 4.88), respectively. In PG, the AD values for ESAS acquired the 256, 1024, 2048, and 8192-points time windows were 1.21 (SD = 5.32), 1.21 (SD = 5.20), 1.07 (SD = 5.13), and 1.93 (SD = 5.32), respectively. In AG, the AD values for ESAS the 256, 1024, 2048, and 8192-points time windows were 1.68 (SD = 5.66), 3.43 (SD = 4.93), 3.65 (SD = 5.28), and 3.86 (SD = 5.57), respectively. Regarding the results of the 256, 1024, 2048, and 8192-points time windows, the AD value of SG was significantly lower than that of PG (256 points, *p* < 0.0001; 1024 points, *p* = 0.002; 2048 points, *p* < 0.0001; and 8192 points, *p* < 0.0001) and AG (256 points, *p* < 0.0001; 1024 points, *p* < 0.0001; 2048 points, *p* < 0.0001; and 8192 points, *p* < 0.0001) (Fig. 6).

At the 8192-point time window, 208 of 348 samples in the case could be analyzed for all of the expiratory durations. On the other hand, only four samples were analyzed for the entire expiratory duration at the 2048-point window, and no sample was able to cover the entire expiratory duration at the 1024- and 256- point time windows.

## Discussion

Our research team focused on swallowing and respiratory sounds for diagnosing oropharyngeal dysphagia. In a series of our studies, methodology for detecting swallowing sounds was established^9^ and symmetry, reproducibility, and production sites of swallowing sounds were investigated^19,20^. Acoustic analysis of swallowing sounds was performed for examining the physiology of swallowing events or oropharyngeal dysphagic conditions by our research team and other researchers^21–24^. Effectiveness of acoustic and auditory characteristics of swallowing and expiratory sounds for detecting oropharyngeal dysphagia was evaluated by our research team^17,18,25^. However, former studies focusing on respiratory or expiratory sounds as a clinical tool for detecting oropharyngeal dysphagia were lacking in elucidating the acoustic characteristics of respiratory or expiratory sounds.

This study was conducted to clarify the acoustic characteristics of voluntary expiratory sounds by evaluating the maximum amplitude and duration of the expiratory sounds produced before and after swallowing attempts during VF. These acquired data were compared among three groups divided based on VF findings: safe swallow (SG), penetration (PG), and aspiration (AG). Frequency characteristics of the voluntary expiratory sounds were also evaluated using AD values and compared among SG, PG, and AG. Therefore, both the time- and frequency-domain characteristics of voluntary expiratory sounds produced before and after swallowing attempts during VF were obtained and compared among the groups.

The maximum amplitude in SG was significantly smaller than those in PG and AG and the mean duration in SG was significantly longer than those in PG and AG in this study. Dysphagic patients are thought to have difficulty in exhalation compared with normal subjects. A previous study showed that it was difficult for dysphagic patients to blow up an 80-cm-long party horn perfectly because of the close relationship between respiratory and swallowing functions^26^.

We used four separate time windows using the Hamming window to identify the frequency bandwidth and target time for analysis. Among the different time windows, the 256-point window showed the most significant inequality of AD values between SG and PG/AG, with an analysis time of 0.032 s. These results suggest that using a shorter analyzing time and including the maximum the wave pattern were important factors. However, there were significant differences in AD values between SG and PG/AG in the other window sizes as well. One of the possible reasons could be that windowing is not important for the low frequency band. It was thought that the duration of most acoustic samples used in this study were over 1 s, and 256 (0.032 s), 1024 (0.128 s), and 2048 (0.256 s) points were too short to analyze the sounds in detail. In this study, the entire expiratory sounds could be analyzed in 208 samples in 8192-point windows. Therefore, the best window size was 8192 points to perform frequency analysis in detail.

There were no significant differences in the AD values between the pre- and post-swallowing expiratory sounds of the SG and PG/AG in this study. Since the analysis was performed by a narrow band using FFT, it was possible to detect low frequencies that cannot be judged by human sensory correction characteristics. It is thought that because the residual secretions in the pharynx and larynx of subjects were cleared, either by strong voluntary cough or by forced expiration, before VF and analysis by FFT, AD values did not show significant differences between pre- and post-swallowing expiratory sounds between SG and PG/AG. This suggests that FFT had the ability to analyze expiratory sounds objectively. AD values acquired in this study might be a key to differentiate between dysphagic oropharyngeal condition and normal condition. The voluntary expiratory sounds were also verified as a useful target for detecting oropharyngeal dysphagia.

Further research is required for obtaining a more incisive discriminant index than the AD value for distinguishing between dysphagic and normal conditions. A large amount of expiratory sound data and sophisticated analyzing methods are needed to meet this requirement.

## Conclusion

Analysis of expiratory sounds during swallowing using FFT provides a more detailed analysis of the low-frequency band compared to analysis using human auditory results. When analyzing the pre- and post-swallowing expiratory sounds using FFT, the frequency band of expiratory sounds was significantly lower in patients in PG/AG than in SG. The results suggested that the AD value detects penetration or aspiration and can be useful in screening for dysphagia. Among the time window sizes used in this study, the most effective was the 8192-point window when the sampling rate was 8000 Hz.
